# Modeling and correction of SCID-X1 using CRISPR-Cas9 homology-directed repair in human HSPCs

**DOI:** 10.1016/j.omtn.2026.102941

**Published:** 2026-04-27

**Authors:** Orli Knop, Nimrod Ben Haim, Nechama Kalter, Michael Rosenberg, Dor Breier, Katia Baider, Yu Nee Lee, Etai Adam, Ido Somekh, Arnon Nagler, Raz Somech, Ayal Hendel

**Affiliations:** 1 Institute of Nanotechnology and Advanced Materials, The Mina and Everard Goodman Faculty of Life Sciences, Bar-Ilan University, Ramat-Gan, Israel; 2 The Division of Hematology and Bone Marrow Transplantation, Chaim Sheba Medical Center, Tel-Hashomer, Ramat Gan, Israel; 3 Sackler Faculty of Medicine, Tel Aviv University, Tel Aviv, Israel; 4 Pediatric Department A and the Immunology Service, Jeffrey Modell Foundation Center, Edmond and Lily Safra Children’s Hospital, Sheba Medical Center, Ramat Gan, Israel; 5 Department of Pediatric Hematology, Oncology, and Bone Marrow Transplant, Edmond and Lily Safra Children’s Hospital, Sheba Medical Center, Ramat Gan, Israel

**Keywords:** MT: RNA/DNA Editing, SCID-X1, IL2RG, CRISPR-Cas9, rAAV6, HSPCs, genome editing, gene replacement

## Abstract

X-linked severe combined immunodeficiency (SCID-X1) is a severe primary immunodeficiency caused by mutations in the *IL2RG* gene, a shared subunit of cytokine receptors critical for the development and function of T and natural killer (NK) cells. The standard treatment, allogeneic hematopoietic stem cell transplantation (HSCT), requires a compatible donor and is often associated with significant transplant-related complications. We aimed to develop a more robust and universal gene therapy by *ex vivo* modification of patient hematopoietic stem and progenitor cells (HSPCs) using the CRISPR-Cas9/rAAV6 gene-editing platform. To evaluate efficiency, we utilized a feeder-free, *in vitro* platform enabling T and NK cell differentiation from modified HSPCs. We investigated two approaches: the cut-site method (inserting a corrective cassette downstream of the start codon within the *IL2RG* locus) and a replacement method (replacing the entire *IL2RG* gene under endogenous regulation). We demonstrated that the cut-site insertion strategy is preferable for SCID-X1 correction, achieving superior homology-directed repair rates, lower toxicity, and significantly improved T and NK cell differentiation, based on phenotypic marker expression. Importantly, corrected patient-derived HSPCs from two SCID-X1 patients, modified using the cut-site approach, successfully demonstrated phenotypic evidence of T cell differentiation. Therefore, our findings firmly establish the feasibility of the cut-site strategy as a universal, high-efficiency, therapeutic solution for SCID-X1.

## Graphical abstract

**Figure:**
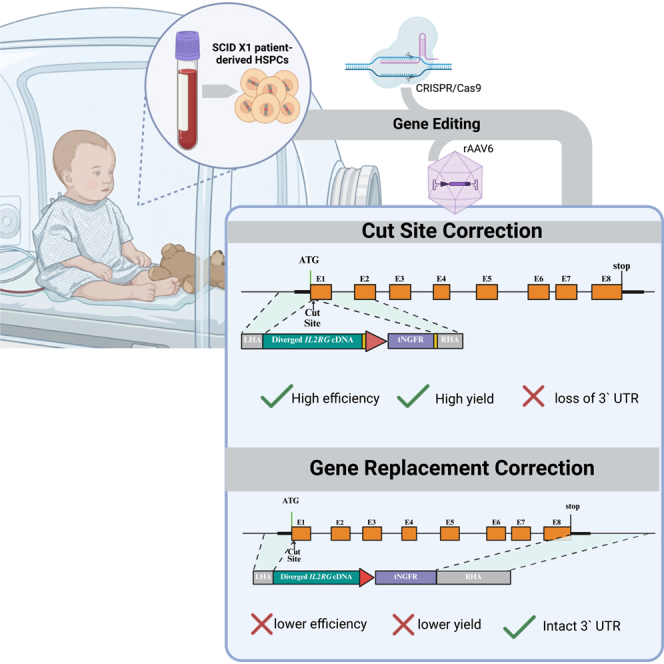

## Teaser

Knop and colleagues use CRISPR-Cas9 HDR editing in human HSPCs to model and correct SCID-X1. They show that cut-site IL2RG correction outperforms gene replacement and restores lymphoid differentiation in patient-derived cells within a feeder-free *in vitro* platform.

## Introduction

Severe combined immunodeficiency (SCID) is a rare and life-threatening disorder caused by defects in T, B, or natural killer (NK) lymphocytes.^1^ The most common SCID, X-linked SCID (SCID-X1),^2^ results from mutations in the interleukin-2 receptor subunit gamma (*IL2RG*) gene, which encodes the γc subunit shared by multiple interleukin receptors involved in T and NK cell differentiation and function.^3^ As a result, SCID-X1 patients exhibit a T− B+ NK− phenotype and are highly susceptible to infections, with even mild illnesses posing lethal risks.^4^ Early diagnosis through newborn screening can prevent unfavorable outcomes.^5^ The conventional treatment for SCID-X1 is hematopoietic stem cell transplantation (HSCT),^6^ which, although efficient, necessitates finding a matched donor and often results in poor immune reconstitution and graft-versus-host disease.^7^ One potential therapeutic approach involves *ex vivo* gene modification of the patient’s own CD34^+^ hematopoietic stem and progenitor cells (HSPCs), followed by autologous reinfusion.^8^ Since few corrected CD34^+^ HSPCs can fully reconstitute the immune system, gene therapy is an appealing solution for SCID-X1.^8^^,^^9^ First-generation therapy used gammaretroviral vectors to insert an *IL2RG* transgene, albeit some patients developed T cell leukemia due to insertional activation of proto-oncogenes via viral integration in the vicinity.^10^^,^^11^^,^^12^^,^^13^ Subsequent efforts tested safer gammaretroviral and lentiviral vectors with removed enhancer sequences,^12^ yet genotoxicity risks persist from semi-random integration and continuous transgene expression, which can result in partial correction, dysregulated gene expression, and toxicity.^14^ Unlike viral vectors, specific gene integration can be achieved via programmable nucleases, such as clustered regularly interspaced short palindromic repeats (CRISPR)-Cas9. An effective strategy for gene editing (GE) is to combine CRISPR-Cas9 for site-specific double-strand break (DSB) generation with recombinant adeno-associated virus serotype 6 (rAAV6) as the DNA donor template, due to its high homology-directed repair (HDR) rates and low off-target integration.^15^^,^^16^^,^^17^^,^^18^ Nevertheless, cellular DNA repair proteins detect rAAV6 vectors and induce DNA damage response, resulting in a certain level of toxicity.^19^ Recent studies using the CRISPR-Cas9/rAAV6 system for SCID-X1 correction demonstrated successful *IL2RG* integration, promoting functional T and NK cell development and engraftment in immunodeficient mice.^20^^,^^21^ In this study, we established a feeder-free, *in vitro* platform for modeling, disruption, and correction of SCID-X1 in healthy male CD34^+^ HSPCs. This system enables (1) testing GE strategies on healthy cells, rather than relying on scarce patient cells and (2) *in vitro* differentiation (IVD) to both T and NK cells. Using this system, we developed two SCID-X1 correction strategies: a cut-site (CS) DNA donor for inserting a corrective cassette near the *IL2RG* start codon, and a gene-replacement (GR) DNA donor to replace the entire *IL2RG* coding sequence (CDS). Our results indicate that the CS strategy is preferable, showing lower toxicity, higher HDR rates, and improved *in vitro* T cell differentiation (IVTD) and *in vitro* NK cell differentiation (IVNKD). Furthermore, applying the CS strategy to patient-derived HSPCs restored IVTD.

## Results

### Modeling SCID-X1 in CD34^+^ HSPCs using the CRISPR-Cas9/rAAV6 system

**Figure 1:**
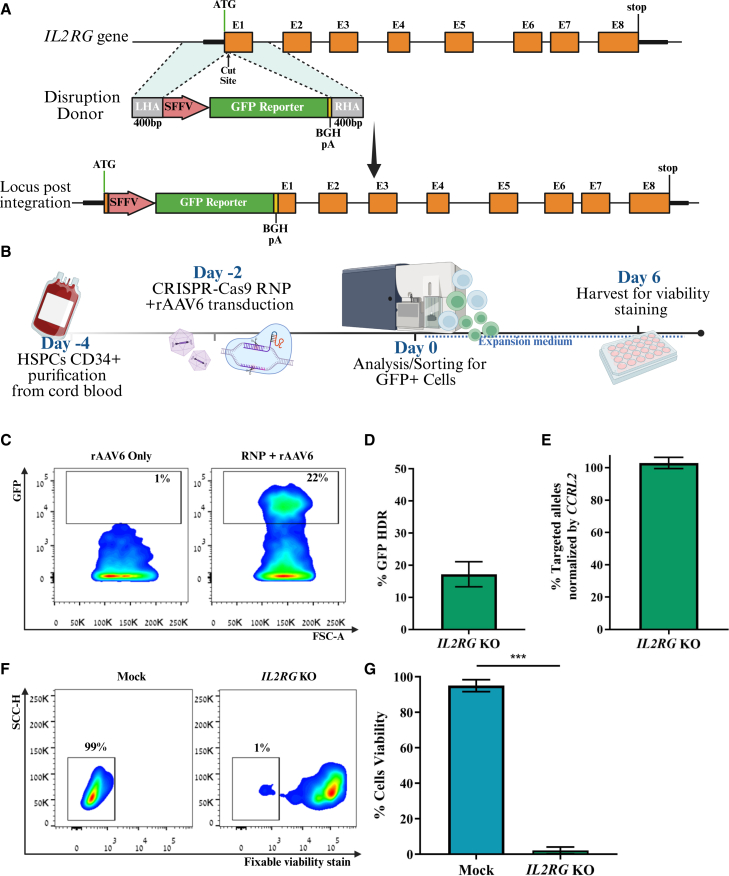
SCIDX-1 modeling in CD34^+^ HSPCs (A) Schematic of *IL2RG* disruption strategy using a GFP donor containing HAs and a pA sequence, resulting in halting the transcription of the endogenous *IL2RG* gene along with insertion of the reporter downstream of the start codon. (B) Schematic of the IVD assay. (C) Representative FC plots 2 days post-electroporation following CRISPR-Cas9/rAAV6 or rAAV6-only transduction. (D) GFP mono-allelic targeting frequencies at the *IL2RG* locus (*n* = 5). (E) HDR frequencies in the GFP^+^-enriched population as determined by ddPCR. (F) Representative FC plots of fixable viability staining analysis on day 6 following *IL2RG* disruption. (G) Cell death frequencies measured across independent differentiation experiments (*n* = 3). Data are presented as mean ± SEM; ∗∗∗*p* < 0.001; *n* = 5.

To establish a functional model of *IL2RG* loss and recapitulate the SCID-X1 phenotype in the IVD system, we knocked out the *IL2RG* gene. We designed a rAAV6 *IL2RG* disruption DNA donor as a template for HDR-mediated integration. The donor included a GFP reporter cassette under the SFFV promoter with a BGH polyadenylation (pA) signal, flanked by 400 bp homology arms (HAs) targeting a CS downstream of the ATG codon^16^^,^^22^ (Figure 1A). Next, we edited cord blood (CB) CD34^+^ HSPCs from healthy donors with a 4 μM ribonucleoprotein (RNP) complex (Figure 1B). The HDR editing efficiency was 17.2% (Figures 1C and 1D). Since SCID-X1 is an X-linked disease, all experiments were conducted using male cells, requiring the editing of a single allele. We then sought to examine the effects of disruption of the *IL2RG* gene on IVTD and IVNKD. Sorted GFP+ cells were seeded in a T- and NK-lineage IVD assay. Droplet digital PCR (ddPCR) analysis verified that the sorted cells contained the complete knockout (KO) disruption (Figure 1E). The *IL2RG* mono-allelic modified cells did not survive the early stages of IVD toward T and NK progenitor cells, mimicking the SCID-X1 phenotype (Figures 1F and 1G). This model provides a platform to assess the impact of *IL2RG* disruption on lymphoid differentiation and serves as a basis for subsequent gene correction experiments.

### Developing CRISPR-Cas9 HDR strategies to correct SCID-X1

**Figure 2:**
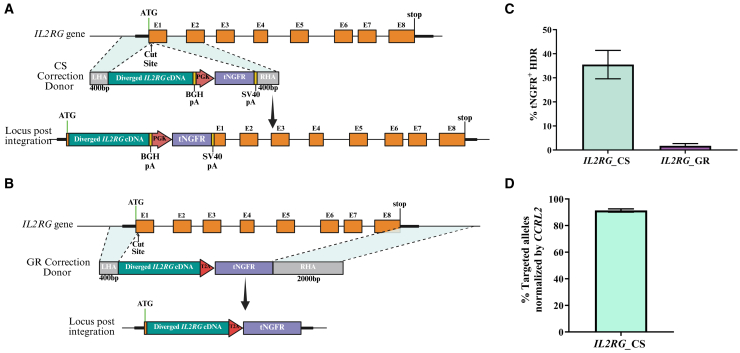
HDR-mediated targeted integration of the *IL2RG* corrective transgene (A) Schematic of the *IL2RG* CS correction donor containing diverged *IL2RG* cDNA, followed by a pA sequence, homology arms (HAs), and a tNGFR reporter cassette. The modified *IL2RG* locus following integration is shown below. (B) Schematic of the IL2RG GR correction donor containing diverged *IL2RG* cDNA and a T2A-tNGFR reporter cassette with upstream and downstream HAs. (C) tNGFR HDR frequencies in CD34^+^ HSPCs 2 days following CRISPR-Cas9/rAAV6 editing, measured by FC (CS, *n* = 4; GR, *n* = 4). (D) Site-specific HDR efficiencies at the *IL2RG* locus measured by ddPCR following enrichment of tNGFR^+^ cells (CS, *n* = 4). Data are presented as mean ± SEM.

Since SCID-X1 is caused by various *IL2RG* mutations, we aimed to create a universal correction strategy applicable to all patients. We developed a corrective rAAV6 DNA donor with a codon-wobbled *IL2RG* cDNA containing silent mutations to preserve the protein sequence while reducing similarity to the genomic sequence to avoid recognition as HA. For enrichment of the edited cell population, a corrective rAAV6 DNA donor was designed to include a truncated human nerve growth factor receptor (tNGFR) reporter cassette, which has been previously approved for clinical applications.^18^ This donor, with 400 bp HAs flanking the Cas9-induced CS, enables targeted insertion (termed *IL2RG*_CS) (Figure 2A). The CS strategy knocks in a corrected cDNA *IL2RG* under a synthetic SV40 pA element instead of the endogenous 3′ UTR, potentially altering native expression. We hypothesized that a correction strategy preserving the endogenous regulation could replicate physiological protein expression levels and improve the immune system reconstitution. We, therefore, developed a second donor to replace the full *IL2RG* gene (*IL2RG*_GR). Based on prior findings that longer HAs enhance HDR rates,^23^ the GR vector included a 400 bp left HA near the CS and a 2,000 bp right HA downstream of the *IL2RG* stop codon, preserving 5′ and 3′ regulatory elements (Figure 2B).

To evaluate HDR integration frequency, CD34^+^ HSPCs were electroporated with the *IL2RG*-RNP complex and transduced with either CS or GR DNA donors. HDR efficacy was determined by tNGFR expression analysis via flow cytometry (FC), showing markedly higher integration in *IL2RG*_CS-edited cells (35%) versus *IL2RG*_GR-edited cells (2%) (Figure 2C). Subsequent ddPCR analysis of tNGFR-positive *IL2RG*_CS-edited cells indicated successful editing in 92% of alleles (Figure 2D).

### CS vs. GR correction strategies: Differentiation of edited CD34^+^ HSPCs into T cell lineages

**Figure 3:**
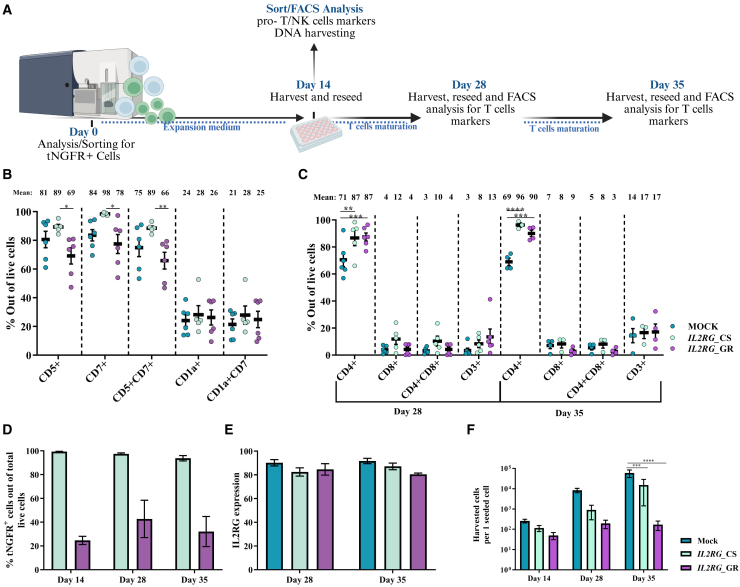
Comparison of CS and GR correction strategies during T-lineage differentiation of edited CD34^+^ HSPCs (A) Schematic of the feeder-free IVTD assay. (B) FC analysis at day 14 showing expression of CD7, CD5, and CD1a (mock, *n* = 6; CS, *n* = 5; GR, *n* = 6). (C) FC analysis at days 28 and 35 showing expression of CD4, CD8, and CD3 (mock: day 28, *n* = 6; day 35, *n* = 4; CS: day 28, *n* = 5; day 35, *n* = 3; GR: day 28, *n* = 6; day 35, *n* = 4). (D) FC analysis of tNGFR expression during differentiation (CS: day 14, *n* = 5; day 28, *n* = 5; day 35, *n* = 3; GR: day 14, *n* = 6; day 28, *n* = 6; day 35, *n* = 4). (E) FC analysis of IL2RG expression at days 28 and 35 (mock: day 28, *n* = 6; day 35, *n* = 3; CS: day 28, *n* = 5; day 35, *n* = 3; GR: day 28, *n* = 6; day 35, *n* = 3). (F) Cell yield per seeded cell during differentiation (mock: day 14 and 28, *n* = 9; day 35, *n* = 5; CS: day 14 and 28, *n* = 8; day 35, *n* = 4; GR: day 14, *n* = 5; day 28, *n* = 6; day 35, *n* = 4). Data are presented as mean ± SEM; ∗*p* < 0.05, ∗∗*p* < 0.01, ∗∗∗*p* < 0.001, ∗∗∗∗*p* < 0.0001 (two-way ANOVA).

We next evaluated the potential of *IL2RG*-edited CD34^+^ HSPCs to differentiate into T cell populations. For the CS strategy, edited CD34^+^ HSPCs were enriched for tNGFR^+^ cells (Figure S5A) and cultured in an IVD assay, while GR-edited cells were seeded directly due to the very low frequency of HDR integration, which resulted in insufficient numbers of reporter-positive cells for reliable sorting while maintaining adequate cell numbers for downstream differentiation assays. After 14 days, cells were analyzed for CD5, CD7, and CD1a T cell progenitor markers. At this stage, cells were sorted based on CD7 expression to enrich for a lymphoid-committed population, thereby reducing non-lymphoid contamination (Figure S5B). Next, the cells were tested for T cell markers (CD3, CD4, and CD8) (Figure 3A; Figure S5C).

By day 14 of differentiation, *IL2RG*-edited cells had differentiated into pro-T cells, as indicated by high levels of CD7^+^ and CD5^+^ markers, though GR-edited cells had lower CD5 and CD7 expression than CS-edited cells (Figure 3B; Figure S1A). By days 28 and 35, cells matured into T cell populations, including single-positive (SP) CD4^+^ and SP CD8^+^ cells, as well as the double-positive (DP) CD4^+^CD8^+^ population. Interestingly, the CD4^+^ population was higher in both CS- and GR-edited cells compared to mock (day 28: mock-71%, CS-87%, GR-87%; day 35: mock-69%, CS-96%, GR-90%) (Figure 3C; Figure S1B). This observation may reflect altered IL2RG expression dynamics in the corrected cells, which could influence cytokine signaling pathways involved in T cell differentiation.

Through differentiation, tNGFR levels in CS-sorted cells remained consistent, indicating survival and proliferation of edited cells. In contrast, tNGFR expression in GR-edited cells, which were seeded without enrichment, increased from day 0 (2%) through days 14 (25%), 28 (43%), and 35 (32%), suggesting negative selection against NHEJ-repaired cells with insertions or deletions (indels) that disrupted normal IL2RG production (Figure 3D).

To examine whether the GE strategies restore physiological protein levels, we measured IL2RG expression in the edited cells on days 28 and 35. Both CS- and GR-edited cells exhibited high IL2RG expression (80%–90%) (Figure 3E). Nevertheless, IL2RG mean fluorescence intensity (MFI) was higher in GR cells than CS cells, while maximal intensity was observed in mock cells (Figure S1C). Although all cell groups exhibited similar viability on day 14, viability was reduced by days 28 and 35, and cell yield was lower in edited cells, likely due to rAAV6 toxicity. Notably, GR cells had a lower yield than CS cells (Figure 3F; Figure S1D).

### Differentiation of edited CD34^+^ HSPCs into NK lineages

**Figure 4:**
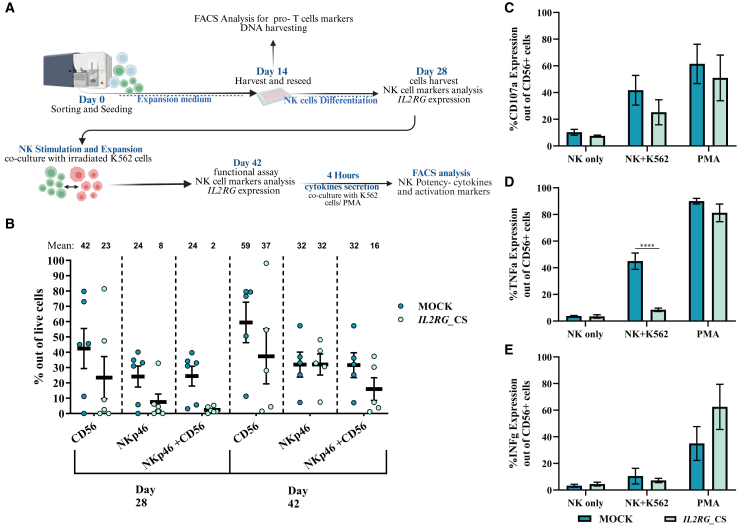
Differentiation of edited CD34^+^ HSPCs into NK lineages (A) Schematic of the feeder-free *in vitro* NK cell differentiation (IVNKD) assay. (B) FC analysis of NK marker expression (CD56 and NKp46) at days 28 and 42 (mock: day 28, *n* = 8; day 42, *n* = 6; CS: day 28, *n* = 5; day 42, *n* = 5). (C–E) FC analysis of NK cell functional markers following 42 days of differentiation and stimulation with K562/PMA, including (C) CD107a, (D) TNF-α, and (E) IFNγ (mock, *n* = 4; CS, *n* = 4). Data are presented as mean ± SEM; ∗∗∗∗*p* < 0.0001 (two-way ANOVA).

Since SCID-X1 is also characterized by impaired NK development, we investigated whether CS and GR correction strategies promote NK-lineage development and restore features associated with NK cell phenotype and activity. HSPCs were subjected to T lymphoid differentiation for 14 days. At this time point, the culture contains both pro-T cells and common lymphoid progenitors (CLPs), the precursors of T and NK cells, respectively. CD7-expressing cells were then sorted and reseeded in conditions supporting the differentiation of CLPs into NK cells (Figure 4A; Figure S5D).

By day 28, NK cell lineages were evident in both mock and CS-edited cells, although differentiation was more robust in mock cells, particularly among NKp46 subpopulations (mock: 24%, *IL2RG*_CS: 8%) (Figure 4B; Figure S2A). In GR-edited cells, tNGFR expression was negligible, and the tNGFR^+^ cells lacked NK markers; thus, only CS-edited cells were analyzed further. On day 42, after K562 stimulation, NKp46+ expression in CS-edited cells increased to 32% (Figure 4B). While IL2RG levels were similar, mock cells showed higher IL2RG MFI than CS-edited cells (Figures S2B and S2C). Furthermore, tNGFR levels remained high throughout the differentiation (Figure S2D). Additionally, the cellular yield of CS-edited cells was lower compared to mock cells, despite similar viability (Figures S2E and S2F). These results suggest that while differentiation of CS-edited cells is slower, they can effectively support NK-lineage differentiation.

Next, we assessed the cytotoxic potential of edited CD34-derived NK cells after 42 days. The analysis focused on the CD56^+^ population in both mock and *IL2RG*_CS groups post-incubation with K562/PMA (Figure S3A). Following PMA stimulation, both groups demonstrated comparable levels of CD107a, TNF-α, and IFNγ. However, mock cells co-cultured with K562 displayed higher cytotoxic activity than *IL2RG*_CS cells, which had reduced CD107a and TNF-α expression (Figures 4C–4F; Figure S3B). Overall, the edited cells showed an immune response against cancer cells, albeit with less efficacy.

### CRISPR-mediated GE rescues IVTD in SCID-X1 patient-derived HSPCs

**Figure 5:**
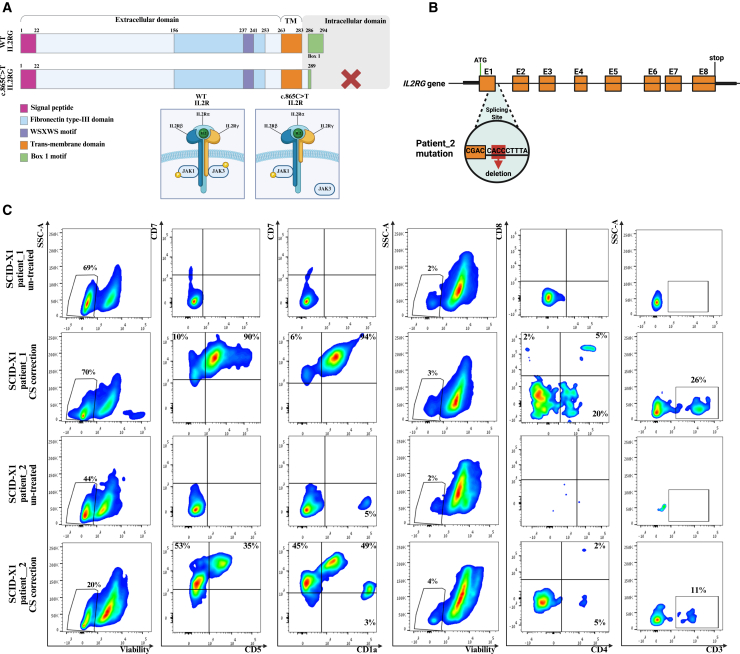
SCID-X1 patient-derived HSPC correction rescues T cell differentiation (A) A representative illustration of the *IL2RG* transcript and WT *IL2R* compared to c.865C>T SCID-X1 patient_1. (B) A representative illustration of the *IL2RG* gene and the mutation of SCID-X1 patient_2 (c.115 + 2_115+4del). (C) FC analysis on days 14 and 28 of IVTD. On day 14, the cells were tested for viability and pro-T cell markers (CD7, CD5, and CD1a). On day 28, cells were tested for viability and T cell markers (CD3, CD4, and CD8). The upper and lower images show the results of SCID-X1 patient_1 and patient_2, respectively.

Following the modeling of SCID-X1 correction in healthy donor HSPCs, we obtained peripheral blood (PB) samples from two male infants diagnosed with SCID-X1 [T-cell receptor excision circles (TRECs) = 0] via newborn screening. The patients demonstrated a low percentage of T cells, an elevated percentage of B cells, and an unexpected presence of NK cells (Figure S4A). The first patient had a hemizygous nonsense mutation (c.865C>T; p. Arg289Ter) in the *IL2RG* gene, resulting in a premature stop codon. This mutation leads to the production of a truncated membrane-bound protein lacking the intracellular domain critical for JAK3 binding, which results in impaired T cell differentiation^24^^,^^25^^,^^26^ (Figure 5A). The second patient had a previously unreported hemizygous deletion mutation (c.115+2_115+4del) in intron 1 of the *IL2RG* gene, which may lead to disruption of mRNA splicing and result in a defective protein (Figure 5B).

To examine the CS correction strategy for SCID-X1, we edited patient-derived HSPCs and evaluated their lymphoid differentiation potential. PB HSPCs from patients were edited with the *IL2RG* RNP complex and CS rAAV6 DNA donor. Untreated and corrected HSPCs were seeded for IVTD. Notably, high cell mortality was observed in both groups on day 28, likely due to the mutant phenotype in untreated cells and rAAV6 toxicity^19^^,^^27^^,^^28^ in the corrected cells (Figure 5C). While the *IL2RG* KO model demonstrated high mortality by day 6 (Figure 1A), untreated cells from both patients remained viable by day 14, possibly due to the residual activity of IL2RG as evidenced by the presence of NK cells in the patients (Figure 5C; Figure S4A).

On day 14 of differentiation, corrected cells from both SCID-X1 patients showed higher expression of pro-T cell markers compared to untreated cells (Figure 5C). Furthermore, on day 28, while untreated cells failed to mature into T cells, corrected cells from both patients demonstrated phenotypic differentiation toward T cell lineages, as reflected by the expression of CD4 and CD3 markers. Moreover, by day 28, presumably following positive selection, the proportion of edited cells (tNGFR^+^ cells) of SCID-X1 patient_2 increased to 98% (Figure S4B).

These results indicate that, despite high cell mortality during IVD, applying the CRISPR-Cas9/rAAV6-mediated correction protocol on CD34^+^ HSPCs derived from both patients resulted in a successful rescue of SCID-X1 phenotypes.

### Assessing the off-target profile of the *IL2RG* gRNA

A known limitation of the CRISPR-Cas9 strategy is the risk of unintended DSBs at off-target sites (OTSs), potentially leading to genotoxic effects.^29^ To evaluate *IL2RG* single-guide RNA (sgRNA) safety, we conducted genome-wide, unbiased identification of DSBs enabled by sequencing (GUIDE-seq) in an HEK293-Cas9 overexpression system, highly sensitive for OTS detection.^30^ We identified 24 potential OTSs, all of which were located within introns or non-coding regions (Table S7).

**Figure 6:**
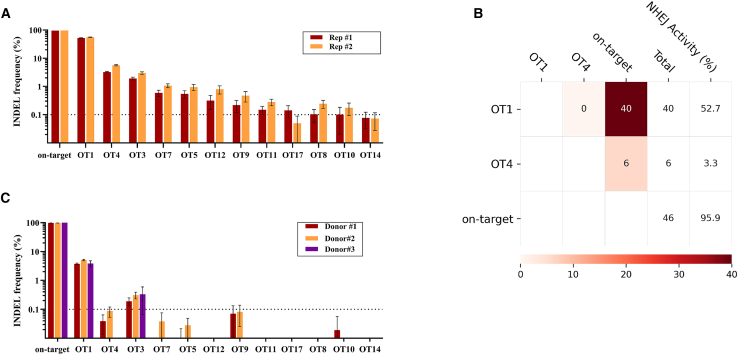
Off-target analysis of *IL2RG* sgRNA (A) rhAmpSeq analysis of off-target editing in HEK293-Cas9 cells (two independent replicates). Sites with editing frequencies above the 0.1% detection threshold are shown. Data are presented as mean ±95% confidence intervals. (B) rhAmpSeq-based analysis of translocation events in HEK293-Cas9 cells. Translocation frequencies between target sites are shown. NHEJ activity at each site is indicated. (C) rhAmpSeq analysis of off-target editing in CD34^+^ HSPCs at the sites identified in (A) (*n* = 3). Data are presented as mean ±95% confidence intervals.

Editing levels at GUIDE-seq-identified OTSs were precisely quantified using an rhAmpSeq assay.^30^ HEK293-Cas9 cells edited with *IL2RG* guide RNA (gRNA) showed one predominant OT1 with a 50% indel rate and several sites with indel rates below 5% (Figure 6A). Furthermore, we detected translocations between the on-target site and two OTSs: OT1 (40 reads) and OT4 (6 reads) (Figure 6B). Subsequently, we repeated the analysis in CD34^+^ HSPCs from three healthy CB donors edited with the RNP complex. We observed a substantial reduction in the off-target profile in the CD34^+^ HSPCs. Only OT1 and OT3 showed low indel activity (<10% and <1%, respectively), with no chromosomal translocations observed (Figure 6C).

## Discussion

This study presents two GE approaches for SCID-X1 correction: insertion of a corrective cassette at the CS or full CDS replacement. Both strategies are performed *ex vivo*, require minimal input cells, and can be effectively evaluated via NK and T cell IVD assay. We demonstrated that CD34^+^ HSPCs edited with either CS or GR strategy developed into cells expressing T cell markers with sufficient γc expression, with the CS strategy yielding a higher extent of NK differentiation and proliferation. Notably, patient-derived HSPCs were corrected with the CS donor differentiated to T cells *in vitro*, underscoring the feasibility of SCID correction in a small number of PB-derived cells.

The *in vitro* system facilitates GE strategy development and testing in healthy HSPCs, bypassing the scarcity of patient cells. We implemented a GE model based on Iancu et al.,^18^ which targeted somatic recessive diseases requiring disruption of both alleles to replicate disease characteristics. In contrast, SCID-X1’s X-linked nature allows single-allele editing in male donor cells, simplifying the design. Our system assesses IVTD and IVNK from edited CD34^+^ HSPCs, both affected in SCID-X1. Nonetheless, our feeder-free platform requires no stromal cells and derives T and NK progenitors from the same edited cells.

The CS strategy only partially preserves endogenous regulation, distancing 3′ UTR elements and positioning *IL2RG* cDNA under an artificial SV40 pA element. Therefore, we evaluated a GR approach to preserve native locus structure for more physiological expression, as previously suggested.^23^^,^^31^ Nonetheless, the CS approach was superior to the GR approach, yielding higher cell counts and enabling NK compartment reconstitution. Nevertheless, the edited NK cells exhibited reduced differentiation and reactivity toward target cells compared to control cells. This may also influence broader differentiation dynamics, as reflected by the increased proportion of CD4^+^ and CD8^+^ cells observed in the corrected populations. Of note, lentiviral GE studies in rodents and humans have shown similar challenges with NK reconstitution,^32^ likely due to the higher IL2RG expression required for NK cell development compared to T cells.^33^^,^^34^ Insufficient transgene expression in both gene-correction approaches likely restricted NK cell development, explaining the discrepancies in NK subpopulation ratios between CS and mock cells. Additionally, reduced IVNKD in edited cells can be attributed to sustained stress from viral presence.

rAAV6 toxicity is a major challenge in GE experiments as viral vectors activate the DNA damage response (DDR) pathway.^19^^,^^35^^,^^36^ We observed reduced proliferation and reconstitution in edited cells, particularly in GR cells, possibly due to the GR vector’s ∼30% larger cargo compared to the CS vector, which increases toxicity and impairs proliferation. Additionally, HSPCs from patients' PB were more sensitive to rAAV6-mediated toxicity than CB HSPCs from healthy donors, similar to findings by Castiello et al. in *RAG1*-SCID cells.^37^ In order to address the problem of toxicity, a number of research groups attempted to reduce toxicity by lowering viral load and enhancing HDR rates through the inhibition of alternative repair pathways.^21^^,^^38^^,^^39^^,^^40^ Other studies inhibited DDR activation by targeting p53 and related molecules.^41^ Also, non-viral methods, such as single-stranded oligodeoxynucleotides (ssODNs), offer a valid alternative that minimizes DDR activation.^39^^,^^42^^,^^43^ However, ssODNs lack the capacity for larger genes such as *IL2RG* CDS,^42^ and their implementation in SCID-X1 requires a mutation-specific design. Given SCID-X1’s life-threatening nature, urgent treatment is essential; the universal rAAV6 vector enables immediate treatment post-diagnosis. Alternatively, double-stranded DNA can be used for non-viral delivery of larger templates, despite added cellular toxicity.^43^

We previously reported an exon replacement strategy to correct *RAG2*-SCID, showing similar results to a CS vector in IVTD assay. However, in most disorders, including SCID-X1, mutations are scattered across multiple exons, and a GR strategy that fits all mutations requires replacing the entire gene, exons and introns alike. Such GR strategy restores only a single isoform and disrupts the endogenous structure. Moreover, in the GR strategy, the extended distance between HAs requires arm elongation to achieve efficient HDR rates.^23^^,^^31^ The HA extension increases the donor DNA size, contributing to observed toxicity and limiting available space within rAAV6 vectors.^44^ Therefore, we propose that exon replacement is most effective when the CDS is within a single exon, as with *RAG2* and *RAG1* loci, or when mutations cluster within one exon. For other genes, the CS strategy might be preferable. Given the variable responses of editing machinery across genomic loci and the influence of epigenetic factors,^45^ each gene should be evaluated independently.

In this work, we edited CD34^+^ HSPCs from two sources: healthy CB donors and patient PB donors. While CB offers easy collection, high CD34^+^ yield, and better viability, it is difficult to obtain CB from SCID patients diagnosed post-birth. PB samples are more accessible, and, while limited in volume, mobilizing agents can significantly increase CD34^+^ HSPC yield.^46^ Therefore, our clinical approach involves isolating CD34^+^ cells from mobilized PB or bone marrow, editing *ex vivo*, and transplanting them back into the patient.

The major limitation of this study is the limited availability of patient-derived CD34^+^ HSPCs, which restricted extensive functional assessment of the differentiated immune populations. Nevertheless, previous studies using larger cell numbers have demonstrated functional restoration following SCID correction within the same pipeline,^18^^,^^23^ including the generation of diverse T cell repertoires. Future studies will expand this platform to include comprehensive functional characterization of corrected immune cells. Therefore, the current study primarily focuses on phenotypic assessment of lineage restoration.

In summary, our study highlights the potential of HDR-mediated GE for SCID-X1. Specifically, we demonstrate that, for *IL2RG*, a cDNA insertion at the CS is more effective than gene replacement, enabling T and NK cell reconstitution. Importantly, when applied to two SCID-X1 patient-derived HSPCs, this approach successfully rescued the patient phenotype, indicating potential clinical use for SCID-X1. Notably, our conclusions are based on phenotypic evidence of lymphoid lineage restoration and not on a comprehensive functional characterization of fully mature T or NK cells. While the CS strategy is effective and shows significant potential for clinical applications, further research is warranted to optimize this strategy before contemplating clinical applications.

## Materials and methods

### CD34^+^ HSPC purification

Human CB-derived CD34^+^ HSPCs from male donors were sourced from Sheba Medical Center CB bank under institutional review board-approved protocols. CB donations were collected post-consent from the obstetrics department, without donor compensation. Mononuclear cells were separated from CB by Ficoll density gradient centrifugation using Lymphoprep (Alere Technologies, Norway). CD34^+^ HSPCs were separated using CD34^+^ Microbead Kit Ultrapure (Miltenyi Biotec, Germany) and cultured for 48 h in CD34 SFEM medium (Table S1). CD34^+^ HSPCs from patient PB samples, obtained with consent from Sheba Medical Center under an institutional review board, were isolated by enrichment via RosetteSep (STEMCELL Technologies, Canada) and subsequent loading on Lymphoprep (Alere Technologies, Norway). Due to the lower frequency of CD34^+^ cells in patient-derived PB samples compared to CB, this isolation approach was selected to minimize cell loss and preserve cell yield for downstream experiments. Cell sorting was done 48 h post-editing for CD34/high and tNGFR or CD34/high only. tNGFR was used as a surface reporter to enable the identification and enrichment of edited cells by FC.

### rAAV6 DNA donor design and production

Assembly of rAAV6 DNA donor plasmids was performed using the NEBuilder HiFi DNA Assembly kit (New England Biolabs, USA) into a backbone of pAAV-MCS plasmid containing AAV2-specific inverted terminal repeats. The construction and sequence of the rAAV6 DNA donor are described in Table S2. The rAAV6 vectors were produced by Vector Core in large-scale rAAV6 batches at the University of North Carolina (Chapel Hill, USA).

### CRISPR-Cas9 genome targeting

The modified *IL2RG* sgRNA was synthesized by Integrated DNA Technologies (IDT, USA),^22^ with the target sequence 5′- UGGUAAUGAUGGCUUCAACA-3′. It was complexed with Alt-R Cas9 protein (IDT, Coralville, IA) at a 1:2.5 molar ratio (Cas9: sgRNA).^30^ CD34^+^ HSPCs were electroporated with a final 4 μM concentration using the Lonza Nucleofector 4D (program DZ100) and P3 primary cell solution (Lonza Biosciences). Electroporated cells were transduced with disruption/correction rAAV6 DNA donors at MOIs of 6,250 viral genomes (VG)/cell or 2,500 VG/cell, respectively. Two days post-electroporation, CD34^+^ HSPCs were stained with antibodies (Table S3) and analyzed/sorted by FC for mono-allelic-positive cells. For the second SCID-X1 patient, editing occurred on day 4 after CD34 enrichment using Aria III cell sorter (BD Biosciences). Unedited control (mock) samples consisted of CD34^+^ HSPCs cultured under identical conditions in the absence of CRISPR-Cas9 RNPs or donor templates. These cells were not transduced and served as unedited controls.

### IVTD and IVNKD

StemSpan T and NK cell generation kits (StemSpan Technologies, USA) were used for the expansion and differentiation of edited and untreated CD34^+^ HSPCs to T and NK cells, according to the manufacturer’s protocol. After 14 days, the cells were divided into two systems for differentiation into T and NK cells. Cells were stained on days 14, 28, and 42 with Viability Stain 510 according to the manufacturer’s instructions. Dead cells were identified based on positive staining, and viable cells were defined as negative for the viability dye and used for downstream analysis. Cells were then stained with antibodies listed in Table S3 and analyzed by FC. IL2RG surface expression was assessed by FC within the indicated gated populations. Expression levels were quantified both as the percentage of IL2RG-positive cells and as MFI of the stained population.

### ddPCR assay

Genomic integration quantification of edited *IL2RG* CD34^+^ HSPCs was performed by droplet digital PCR (ddPCR, Bio-Rad). DNA was extracted from cell populations on day 14 of IVTD and IVNKD using GeneJET genomic DNA purification kit (Thermo Fisher Scientific). Concentration (copies/μL) of *IL2RG* site-specific DNA donor integration and wild-type (WT) *CCRL2* alleles was calculated using the QuantaSoft analysis software (Bio-Rad, USA). Data are normalized by targeted *CCRL2* alleles. Primers and probe sequences are presented in Table S4.

### NK cell functional assay

Following 28 days of IVNKD, cells were seeded with irradiated K562 cells for 2 weeks. Cells were then harvested and incubated for 4 h at 37°C, 5% CO_2_, with/without fresh K562 target cells,^47^ as specified in Table S5. NK-like cells were co-cultured with K562 cells in direct contact without prior separation. During FC analysis, NK cells were identified based on CD56 expression, allowing exclusion of K562 target cells from downstream analysis. Harvested cells were stained using the FoxP3 staining buffer set (Miltenyi Biotec, Germany) and intracellular antibodies, according to the manufacturer’s protocol and Table S3.

### Off-target analysis

GUIDE-seq^48^ and rhAmpSeq^49^ experiments were performed as described previously.^19^^,^^30^ GUIDE-seq sequencing data were analyzed using the guide-seq package (v.1.0.2) with default parameters (https://github.com/aryeelab/guideseq). The rhAmpSeq panel design information is located in Table S6. Sequencing data were analyzed using the CRISPECTOR package (v.1.0.5)^50^ with default parameters.

## Data and code availability

Sequencing files of GUIDE-seq and rhAmpSeq experiments were deposited in NCBI BioProject: PRJNA1048126.

## Acknowledgments

We would like to thank the members of the Somech and the Hendel labs for reading the manuscript and providing practical advice. Additionally, we would like to thank D. Russell for providing the pDGM6 plasmid. All figures in this manuscript were created with BioRender.com. This work was supported by the 10.13039/501100003977Israel Science Foundation (10.13039/501100003977ISF)-Israel Precision Medicine Partnership (IPMP) (grant no. 3115/19 to A.H.), 10.13039/501100003977Israel Science Foundation (10.13039/501100003977ISF)-Individual Research Grants (grant no. 2031/19 to A.H.), 10.13039/501100003977Israel Science Foundation (10.13039/501100003977ISF)-Individual Research Grants (grant no. 139/23), and the 10.13039/100001245Jeffrey Modell Foundation (JMF) Translational Research Program.

## Author contributions

O.K., N.B.H., M.R., and D.B. designed and conducted the experiments; N.K. analyzed the GUIDE-seq and rhAmpSeq data; K.B. and A.N. provided CB samples; K.B., I.S., E.A., A.N., and R.S. critically reviewed the experiments and provided important advice; A.H. supervised and conceived the research; and O.K., N.B.H., and N.K. wrote the manuscript, with contributions and input from all authors.

## Declaration of interests

A.H. is the founder and chief scientific officer of Cassidy Bio. Cassidy Bio did not have input into the design, execution, interpretation, or publication of this work.

## Declaration of generative AI and AI-assisted technologies in the writing process

During the preparation of this work, the authors used available AI tools (e.g., ChatGPT) in order to enhance the quality of our writing. After using these tools, the authors reviewed and edited the content as needed and took full responsibility for the content of the publication.

## Supplemental information

Document S1. Figures S1–S5 and Tables S1–S7

Document S2. Article plus supplemental information
