# Supplementary material for: Modeling and correction of SCID-X1 using CRISPR-Cas9 homology-directed repair in human HSPCs
Source: Mol Ther Nucleic Acids. 2026 Apr 27;37(2):102941. doi: 10.1016/j.omtn.2026.102941 (PMC13191621; doi:10.1016/j.omtn.2026.102941)
Supplement: Document S1. Figures S1–S5 and Tables S1–S7 [file mmc1.pdf]

OMTN, Volume *37*

Supplemental information

Modeling and correction of SCID-X1 using CRISPR-Cas9 homology-directed repair in human HSPCs

Orli Knop, Nimrod Ben Haim, Nechama Kalter, Michael Rosenberg, Dor Breier, Katia Baider, Yu Nee Lee, Etai Adam, Ido Somekh, Arnon Nagler, Raz Somech, and Ayal Hendel

Table S1. Composition of the HSPC culture medium

Name	Concentration	Supplier information
SFEM II medium		Stemcell Technologies, Canada
Flt3	100 ng/ml	Peprotech, Israel
TPO	100 ng/ml	Peprotech, Israel
SCF	100 ng/ml	Peprotech, Israel
IL6	100 ng/ml	Peprotech, Israel
penicillin	20 unit/ml	Biological Industries, Israel
streptomycin	20 unit/ml	Biological Industries, Israel
UM171	0.035 mg/µl	Stemcell Technologies, Canada
SR1	0.75 mg/µl	Cayman Chemical, USA

Table S2. rAAV6 donors design and sequences.

***IL2RG* disruption rAAV6 donor**	
**LHA cut site**	gcatggcatagaacggtgatgtcgggggtgggggttcagaacttccattatagaaggtaatgatttagaggagaaggtggttgagaatggtgctagtggtagtgaacagatccttcccaggatctagg<br>tgggctgaggatttttgagtctgtgacactattgtatatccagctttagtttctgtttaccaccttacagcagcacctaatctcctagaggacttagcccgtgtcacacagcacatatttgccacaccctctgta<br>aagccctggtttataaggttctttccaccggaagctatgacAGAGGAAACGTGTGGGTGGGGAGGGGTAGTGGGTGAGGGACCCAGGTTCCTGA<br>CACAGACAGACTACACCCAGGGAATGAAGAGCAAGCGCCATGT
**SFFV Promoter**	CCGATAAAATAAAAGATTTTATTTAGTCTCCAGAAAAAGGGGGGAATGAAAGACCCCACCTGTAGGTTTGGCAAGCTA<br>GCTGCAGTAACGCCATTTTGCAAGGCATGGAAAAATACCAAACCAAGAATAGAGAAGTTCAGATCAAGGGCGGGTACA<br>TGAAAATAGCTAACGTTGGGCCAAACAGGATATCTGCGGTGAGCAGTTTCGGCCCCGGCCCGGGGCCAAGAACAGATG<br>GTCACCGCAGTTTCGGCCCCGGCCCGAGGCCAAGAACAGATGGTCCCCAGATATGGCCCAACCCTCAGCAGTTTCTTAA<br>GACCCATCAGATGTTTCCAGGCTCCCCCAAGGACCTGAAATGACCCTGCGCCTTATTTGAATTAACCAATCAGCCTGCTT<br>CTCGCTTCTGTTCGCGCGCTTCTGCTTCCCGAGCTCTATAAAAGAGCTCACAACCCCTCACTCGGCGCGCCAGTCCTCCG<br>ACAGACTGAGTCGCCCGGG
**GFP**	ATGCCCGCCATGAAGATCGAGTGCCGCATCACCGGCACCCTGAACGGCGTGGAGTTCGAGCTGGTGGGCGGCGGAGAG<br>GGCACCCCCGAGCAGGGCCGCATGACCAACAAGATGAAGAGCACCAAAGGCGCCCTGACCTTCAGCCCCTACCTGCTG<br>AGCCACGTGATGGGCTACGGCTTCTACCACTTCGGCACCTACCCCAGCGGCTACGAGAACCCCTTCCTGCACGCCATCA<br>ACAACGGCGGCTACACCAACACCCGCATCGAGAAGTACGAGGACGGCGGCGTGCTGCACGTGAGCTTCAGCTACCGCT<br>ACGAGGCCGGCCGCGTGATCGGCGACTTCAAGGTGGTGGGCACCGGCTTCCCCGAGGACAGCGTGATCTTCACCGACA<br>AGATCATCCGCAGCAACGCCACCGTGGAGCACCTGCACCCCATGGGCGATAACGTGCTGGTGGGCAGCTTCGCCCGCAC<br>CTTCAGCCTGCGCGACGGCGGCTACTACAGCTTCGTGGTGGACAGCCACATGCACTTCAAGAGCGCCATCCACCCCAGC<br>ATCCTGCAGAACGGGGGCCCCATGTTCGCCTTCCGCCGCGTGGAGGAGCTGCACAGCAACACCGAGCTGGGCATCGTG<br>GAGTACCAGCACGCCTTCAAGACCCCCATCGCCTTCGCCAGATCTCGAGTCTAG
**BGH polyA**	CTCGAGGGCGCGCCccgctgatcagcctcgactgtgccttctagttgccagccatctgttgtttgcccctcccccgtgccttccttgaccctggaaggtgccactcccactgtcctttcctaat<br>aaaatgaggaaattgcatcgcattgtctgagtaggtgtcattctattctggggggtggggtggggcaggacagcaagggggaggattgggaagacaatagcaggcatgctggggatgcggtgggc<br>t
**RHA cut site**	TGAAGCCATCATTACCATTCACATCCCTCTTATTCCTGCAGCTGCCCCTGCTGGGAGTGGGGCTGAACACGACAATTCTG<br>ACGCCCAATGGGAATGAAGACACCACAGCTGgtgggaaatctgggactggagggggctggtgagaagggtggctgtgggaaggggccgtacagagatctggtgc<br>ctgccactggccattacaatcatgtgggcagaattgaaaagtggagtgggaagggcaaggggggagggttccctgcctcacgctacttcttctttctttcttgtttgtttgtttctttctttcttttgaggcaggg<br>tctcactatgttgcctaggctggtctcaaactcctggcctctagtgatcctcctgcctcagcctttcaaagcaccaggattacagacatgagcca
***IL2RG* CS (cut site) correction rAAV6 donor**	
**LHA cut site**	gcatggcatagaacggtgatgtcgggggtgggggttcagaacttccattatagaaggtaatgatttagaggagaaggtggttgagaatggtgctagtggtagtgaacagatccttcccaggatctagg<br>tgggctgaggatttttgagtctgtgacactattgtatatccagctttagtttctgtttaccaccttacagcagcacctaatctcctagaggacttagcccgtgtcacacagcacatatttgccacaccctctgta<br>aagccctggtttataaggttctttccaccggaagctatgacAGAGGAAACGTGTGGGTGGGGAGGGGTAGTGGGTGAGGGACCCAGGTTCCTGA<br>CACAGACAGACTACACCCAGGGAATGAAGAGCAAGCGCCATGT
**IL2RG diverged cDNA**	TAAAACCCAGCCTGCCCTTTACTAGTCTGCTGTTTCTCCAACTCCCTCTGCTGGGAGTGGGGCTGAACACGACCATCCTC<br>ACCCCTAACGGAAACGAGGATACTACCGCCGATTTCTTTCTGACCACCATGCCAACCGATAGCCTGTCTGTCTCAACCCT<br>GCCCCTGCCTGAAGTCCAGTGCTTTGTCTTCAATGTGGAGTATATGAACTGCACCTGGAATAGCTCCTCTGAACCACAGC<br>CCACCAACCTGACACTGCACTACTGGTATAAGAACAGCGACAATGATAAGGTGCAGAAATGCTCCCATTATCTGTTCTC<br>TGAGGAAATCACCAGTGGGTGTCAGCTGCAGAAGAAAGAGATTCACCTGTACCAGACATTTGTGGTCCAGCTGCAGGA<br>CCCTCGGGAACCACGGAGACAGGCCACTCAGATGCTGAAGCTGCAGAACCTGGTCATCCCCTGGGCTCCTGAGAATCTG<br>ACCCTGCATAAACTGAGTGAGTCACAGCTGGAACTGAACTGGAACAATAGGTTCCTGAATCACTGTCTGGAGCATCTGG<br>TGCAGTACCGCACAGACTGGGATCACTCATGGACTGAACAGAGCGTCGACTATCGACATAAGTTTAGCCTGCCATCCGT<br>GGATGGACAGAAAAGGTACACCTTCCGGGTGCGGAGCCGGTTCAACCCACTGTGCGGATCCGCCCAGCACTGGTCTGA<br>GTGGAGTCACCCCATCCATTGGGGGTCAAACACTAGCAAGGAGAATCCTTTCCTGTTTGCCCTGGAAGCTGTGGTCATTT<br>CCGTGGGATCTATGGGCCTGATCATTTCCCTGCTGTGCGTGTACTTCTGGCTGGAGCGGACTATGCCACGAATTCCCACC<br>CTGAAGAACCTGGAGGACCTGGTGACAGAATATCACGGCAACTTCTCCGCCTGGTCAGGGGTCAGCAAAGGACTGGCA

	GAGTCCCTGCAGCCTGATTACTCTGAGCGGCTGTGCCTGGTGTCCGAAATTCCCCCTAAAGGAGGGGCACTGGGAGAAGGACCTGGAGCCTCTCCATGTAACCAGCACTCTCCTTATTGGGCTCCACCTTGTTATACTCTGAAACCCGAAACCTGA
**BGH polyA**	CTCGAGGGCGCGCCccgctgatcagcctcgactgtgccttctagttgccagccatctgttgtttgcccctcccccgtgccttccttgaccctggaaggtgccactcccactgtcctttcctaataaaatgaggaaattgcatcgcattgtctgagtaggtgtcattctattctggggggtggggtggggcaggacagcaagggggaggattgggaagacaatagcaggcatgctggggatgcggtgggct
**hPGK promoter**	ggtaccgaattcccacGGGGTTGGGGTTGCGCCTTTTCCAAGGCAGCCCTGGGTTTGCGCAGGGACGCGGCTGCTCTGGGCGTGGTTCCGGGAAACGCAGCGGCGCCGACCCTGGGTCTCGCACATTCTTCACGTCCGTTCGCAGCGTCACCCGGATCTTCGCCGCTACCCTTGTGGGCCCCCCGGCGACGCTTCCTGCTCCGCCCCTAAGTCGGGAAGGTTCCTTGCGGTTCGCGGCGTGCCGGACGTGACAAACGGAAGCCGCACGTCTCACTAGTACCCTCGCAGACGGACAGCGCCAGGGAGCAATGGCAGCGCGCCGACCGCGATGGGCTGTGGCCAATAGCGGCTGCTCAGCGGGGCGCGCCGAGAGCAGCGGCCGGGAAGGGGCGGTGCGGGAGGCGGGGTGTGGGGCGGTAGTGTGGGCCCTGTTCCTGCCCGCGCGGTGTTCCGCATTCTGCAAGCCTCCGGAGCGCACGTCGGCAGTCGGCTCCCTCGTTGACCGAATCACCGACCTCTCTCCCCAG
**NGFR**	ATGGGGGCAGGTGCCACCGGCCGCGCCATGGACGGGCCGCGCCTGCTGCTGTTGCTGCTTCTGGGGGTGTCCCTTGGAGGTGCCAAGGAGGCATGCCCCACAGGCCTGTACACACACAGCGGTGAGTGCTGCAAAGCCTGCAACCTGGGCGAGGGTGTGGCCCAGCCTTGTGGAGCCAACCAGACCGTGTGTGAGCCCTGCCTGGACAGCGTGACGTTCTCCGACGTGGTGAGCGCGACCGAGCCGTGCAAGCCGTGCACCGAGTGCGTGGGGCTCCAGAGCATGTCGGCGCCGTGCGTGGAGGCCGACGACGCCGTGTGCCGCTGCGCCTACGGCTACTACCAGGATGAGACGACTGGGCGCTGCGAGGCGTGCCGCGTGTGCGAGGCGGGCTCGGGCCTCGTGTTCTCCTGCCAGGACAAGCAGAACACCGTGTGCGAGGAGTGCCCCGACGGCACGTATTCCGACGAGGCCAACCACGTGGACCCGTGCCTGCCCTGCACCGTGTGCGAGGACACCGAGCGCCAGCTCCGCGAGTGCACACGCTGGGCCGACGCCGAGTGCGAGGAGATCCCTGGCCGTTGGATTACACGGTCCACACCCCCAGAGGGCTCGGACAGCACAGCCCCCAGCACCCAGGAGCCTGAGGCACCTCCAGAACAAGACCTCATAGCCAGCACGGTGGCAGGTGTGGTGACCACAGTGATGGGCAGCTCCCAGCCCGTGGTGACCCGAGGCACCACCGACAACCTCATCCCTGTCTATTGCTCCATCCTGGCTGCTGTGGTTGTGGGCCTTGTGGCCTACATAGCCTTCAAGAGGTGGAACAGGGGGATCCTCTAG
**SV40 poly(A)**	AACTTGTTTATTGCAGCTTATAATGGTTACAAATAAAGCAATAGCATCACAAATTTCACAAATAAAGCATTTTTTTCACTGCATTCTAGTTGTGGTTTGTCCAAACTCATCAATGTATCTTA
**RHA cut site**	TGAAGCCATCATTACCATTCACATCCCTCTTATTCCTGCAGCTGCCCCTGCTGGGAGTGGGGCTGAACACGACAATTCTGACGCCCAATGGGAATGAAGACACCACAGCTGgtgggaaatctgggactggagggggctggtgagaagggtggctgtgggaaggggccgtacagagatctggtgcctgccactggccattacaatcatgtgggcagaattgaaaagtggagtgggaagggcaagggggagggttccctgcctcacgctacttcttctttctttcttgtttgtttgtttctttctttcttttgaggcagggtctcactatgttgcctaggctggtctcaaactcctggcctctagtgatcctcctgcctcagcctttcaaagcaccaggattacagacatgagcca

IL2RG GR (gene replacement) correction rAAV6 donor

**LHA cut site**	gcatggcatagaacggtgatgtcgggggtgggggttcagaacttccattatagaaggtaatgatttagaggagaaggtggttgagaatggtgctagtggtagtgaacagatccttcccaggatctaggtgggctgaggatttttgagtctgtgacactattgtatatccagctttagtttctgtttaccaccttacagcagcacctaatctcctagaggacttagcccgtgtcacacagcacatatttgccacaccctctgtaaagccctggtttataaggttctttccaccggaagctatgacAGAGGAAACGTGTGGGTGGGGAGGGGTAGTGGGTGAGGGACCCAGGTTCCTGACACAGACAGACTACACCCAGGGAATGAAGAGCAAGCGCCATGT
**IL2RG diverged cDNA**	TAAAACCCAGCCTGCCCTTTACTAGTCTGCTGTTTCTCCAACTCCCTCTGCTGGGAGTGGGGCTGAACACGACCATCCTCACCCCTAACGGAAACGAGGATACTACCGCCGATTTCTTTCTGACCACCATGCCAACCGATAGCCTGTCTGTCTCAACCCTGCCCCTGCCTGAAGTCCAGTGCTTTGTCTTCAATGTGGAGTATATGAACTGCACCTGGAATAGCTCCTCTGAACCACAGCCCACCAACCTGACACTGCACTACTGGTATAAGAACAGCGACAATGATAAGGTGCAGAAATGCTCCCATTATCTGTTCTCTGAGGAAATCACCAGTGGGTGTCAGCTGCAGAAGAAAGAGATTCACCTGTACCAGACATTTGTGGTCCAGCTGCAGGACCCTCGGGAACCACGGAGACAGGCCACTCAGATGCTGAAGCTGCAGAACCTGGTCATCCCCTGGGCTCCTGAGAATCTGACCCTGCATAAACTGAGTGAGTCACAGCTGGAACTGAACTGGAACAATAGGTTCCTGAATCACTGTCTGGAGCATCTGGTGCAGTACCGCACAGACTGGGATCACTCATGGACTGAACAGAGCGTCGACTATCGACATAAGTTTAGCCTGCCATCCGTGGATGGACAGAAAAGGTACACCTTCCGGGTGCGGAGCCGGTTCAACCCACTGTGCGGATCCGCCCAGCACTGGTCTGAGTGGAGTCACCCCATCCATTGGGGGTCAAACACTAGCAAGGAGAATCCTTTCCTGTTTGCCCTGGAAGCTGTGGTCATTTCCGTGGGATCTATGGGCCTGATCATTTCCCTGCTGTGCGTGTACTTCTGGCTGGAGCGGACTATGCCACGAATTCCCACCCTGAAGAACCTGGAGGACCTGGTGACAGAATATCACGGCAACTTCTCCGCCTGGTCAGGGGTCAGCAAAGGACTGGCAGAGTCCCTGCAGCCTGATTACTCTGAGCGGCTGTGCCTGGTGTCCGAAATTCCCCCTAAAGGAGGGGCACTGGGAGAAGGACCTGGAGCCTCTCCATGTAACCAGCACTCTCCTTATTGGGCTCCACCTTGTTATACTCTGAAACCCGAAACCTGA
**T2A**	GAGGGCAGAGGAAGTCTTCTAACATGCGGTGACGTGGAGGAGAATCCCGGCCCA
**NGFR**	ATGGGGGCAGGTGCCACCGGCCGCGCCATGGACGGGCCGCGCCTGCTGCTGTTGCTGCTTCTGGGGGTGTCCCTTGGAGGTGCCAAGGAGGCATGCCCCACAGGCCTGTACACACACAGCGGTGAGTGCTGCAAAGCCTGCAACCTGGGCGAGGGTGTGGCCCAGCCTTGTGGAGCCAACCAGACCGTGTGTGAGCCCTGCCTGGACAGCGTGACGTTCTCCGACGTGGTGAGCGCGACCGAGCCGTGCAAGCCGTGCACCGAGTGCGTGGGGCTCCAGAGCATGTCGGCGCCGTGCGTGGAGGCCGACGACGCCGTGTGCCGCTGCGCCTACGGCTACTACCAGGATGAGACGACTGGGCGCTGCGAGGCGTGCCGCGTGTGCGAGGCGGGCTCGGGCCTCGTGTTCTCCTGCCAGGACAAGCAGAACACCGTGTGCGAGGAGTGCCCCGACGGCACGTATTCCGACGAGGCCAACCACGTGGACCCGTGCCTGCCCTGCACCGTGTGCGAGGACACCGAGCGCCAGCTCCGCGAGTGCACACGCTGGGCCGACGCCGAGTGCGAGGAGATCCCTGGCCGTTGGATTACACGGTCCACACCCCCAGAGGGCTCGGACAGCACAGCCCCCAGCACCCAGGAGCCTGAGGCACCTCCAGAACAAGACCTCATAGCCAGCACGGTGGCAGGTGTGGTGACCACAGTGATGGGCAGCTCCCAGCCCGTGGTGACCCGAGGCACCACCGACAACCTCATCCCTGTCTATTGCTCCATCCTGGCTGCTGTGGTTGTGGGCCTTGTGGCCTACATAGCCTTCAAGAGGTGGAACAGGGGGATCCTCTAG
**2K RHA stop site**	ACCCCAATCCTCTGACAGAAGAACCCCAGGGTCCTGTAGCCCTAAGTGGTACTAACTTTCCTTCATTCAACCCACCTGCGTCTCATACTCACCTCACCCCACTGTGGCTGATTTGGAATTTTGTGCCCCCATGTAAGCACCCCTTCATTTGGCATTCCCCACTTGAGAATTACCCTTTTGCCCCGAACATGTTTTTCTTCTCCCTCAGTCTGGCCCTTCCTTTTCGCAGGATTCTTCCTCCCTCCCTCTTTCCCTCCCTTCCTCTTTCCATCTACCCTCCGATTGTTCCTGAACCGATGAGAAATAAAGTTTCTGTTGATAATCATCAAAAATGTCTGTCATGGGGGTGGGGAGCAGGGGAAGCCTGGGATGCAATGAGGGTGGGAGGGGAAGAAAAAGGAGTAGTACATTCATGCTGTGGAGGCAGGAGTATTACCAATTTGCTAGAAAGAGTACTGTTCTGGAAACTGACTTTTTTCCAGGTGCCCTGCACTCATTCCAGGGTTTAGTTTAGAGTTAGACTTACAATGGGCTGGGCGTGGTGGCTCACGCCTGTAATCCCAGCACTTTGGGAGGCCAAAGCGGGTGGATCACGAGGTCAGGAGATCGAGACCAGCCTGGCCAACATGGTGAAACCCCGTCTCTACTAAAAATACAAAAATTAGCCAGGCGTGATGGTGTGCTCCTGTAATTCCAGCTACTTGGGAGGCTGAGGCAGGAGAATCGCTTGAACCCAGGAGGTAGAGGATGCAGTGAGCCAAGATTGTGCCACTGCACTCCAGCCTGGGCGACAGAGGGAGACTCCATCTAAAAAAATAAAAAAAATAAAAAAAGTTAGACTTACCATGTTGTCCCCCCTTTCCAACTAATCTTTAACATACCCCACCCAAGTTTTTCTCTCCTTACCCTTACTCCACTTTTTGGCCACACCTGCCACTTCTGACCTCATAATCCCTTAACTGCAGTCTCAGGCCCCACCCCTGGGGATACTGGTTATCCACAACTATCAACAGCTCTCTCCTGCCTGTCCAGCCTGGGCATCCTCAGCCTATGAAATCCATCTGCCTCTCTAGTCCACCAACCCTATAGGCCTATTTGCCCCACACAAGGCCTCTAGAACACCCTCCTTCTGCATGACTCATCAGGGCACTTTGTGAAGAGTGAGGCGATCTTGAGGGCATGTTCATCTTCATCAAACATGGAGGTGAGGGGCAGCACAGAACTAGAGTCTGTGGATGGGGGTGGAGGGATCTGAGGGCAGTGGAGATCACAG

	AAATGGGCCCATGTGTCCCATCCTATCTCTTCTCAGATAATCAGCAGTTTCTGGTCAATACCAACTGTGCTGTCGTCGTG<br>TTGCTGTATTACATCCGCAGTAAAGTAAAGTTGCCTAAAACAAGTGAGGAGCTTAGGGAGGGGACTAATGGCCAAAGG<br>TCAGAAGGAGTTGGGTCATCTCTAACCCCCAAGCCCTCCTCCTGGCAAGAAGGGAGTTCCTCATATCCTTAGAGCTTCCT<br>CCAACCCATTTTGTATAGGATCACTGGCCCCTTTTGCTCCCACTCTCTTTCCTTAGCTGCCCCAATTACCCTGTGATCTCC<br>CTCCAGACACCATCGATTTGTGTGAACAAACGGGGAAAATGAAGATGCTTTTCCTGATGAAGCCCAATCATGCAGAGTA<br>TGCCAGCAAATACCTTACAGCTCGAAGCACCTACTACGTTTGTAAGGTGGAACGTGGGCCACCAGGTAGAGGCTCAGG<br>AGTGTTCCCCAGAAATAGAATAAGGCCTTCAAGATAGATTATGAACCTTATCTTTAAAACAAGGCATCCAAGGCTGGGC<br>ATAGTGGCTCATGCTTGTAATCTCAGCACTTTGAGAGGCCAAGGCGGGAGAATCGATAGAGGGCAGGAGTTTGAGACC<br>AGCCTGGGCAACATAACAAGAGCCTGCCTCCAAAACCAAAACCAAAACCAAAAACAACCAAAACCAAATTAAAACAA<br>AAAACAGAACACCCTCA

Table S3. Antibodies used for immunophenotyping of T-cell and NK-cell differentiation

	antibody	Clone/cat. No	Supplier information
day 0	PE- CD34	561	BioLegend
	APC-tNGFR	ME20.4	BioLegend
day 14	CD1a	BL6	Beckman Coulter, USA
	CD5	UCHT2	BioLegend
	CD7	CD7-6B7	BioLegend
	APC-tNGFR	ME20.4	BioLegend
	Fixable Viability Stain 510		BD Horizon
T-cells differentiation days 28&35	CD3	UCHT1	BioLegend
	CD4	RPA-T4	BioLegend
	CD8	RPA-T8	BD Horizon
	APC-tNGFR	ME20.4	BioLegend
	IL2RG	QA17A55	BioLegend
	Fixable Viability Stain 510	564406	BD Horizon
NK-cells differentiation days 28&42	PE/Cy7-NKp46	9E2	BioLegend
	BV421-CD56	5.1H11	BioLegend
	APC-tNGFR	ME20.4	BioLegend
	IL2RG	QA17A55	BioLegend
	Fixable Viability Stain 510		BD Horizon
Isotype controls	PE/Cy7 Mouse IgG2a κ	400232	BioLegend
	BV-421 Mouse IgG1κ	400158	BioLegend
	PE Mouse IgG1κ	400112	BioLegend
	PE/Cy7 Mouse IgG1κ	400126	BioLegend
	APC-R700 Mouse IgG1κ	564974	BD Horizon
	APC Mouse IgG1k	400122	BioLegend
NK cell functional assay	PE-Cy7-CD107a	REA792	Miltenyi Biotec
	BV421-CD56	5.1H11	BioLegend
	APC-tNGFR	ME20.4	BioLegend
Intracellular antibodies	PE- Anti-TNFα	REA656	Miltenyi Biotec
	FITC- Anti-IFNγ	45-15	Miltenyi Biotec

Table S4. Primers and probes for ddPCR quantification of *IL2RG* gene editing

CCRL2	Forward	GCTGTATGAATCCAGGTCC
	Reverse	CCTCCTGGCTGAGAAAAAG
	Probe	5HEX/TGTTTCCTC/ZEN/CAGGATAAGGCAGCTGT/3IABkFQ
IL2RG Distruption	Forward	GAGGATTGGGAAGACAA
	Reverse	GGGCATAGTGGTCAGGAAG
	Probe	56-FAM/CGT CAG AAT /ZEN/TGT CGT GTT CAG C/3IABkFQ

IL2RG Correction	Forward	GGTGACCAAGTCAAGGAAGAG
	Reverse	CAGAAAGAAATCGGCGGTAGTA
	Probe	56FAM/AGCACCATT/ZEN/CTCAACCACCTTCTCC /3IABkFQ

Table S5. Stimulation conditions for intracellular cytokine staining of NK cells

	NK Only	K562 only	NK+K562	NK + PMA
Cells amount	100,000 Cells	100,000 cells	100,000 NK+ 100,000 K562	100,000 NK
Medium (0.5ml)	V	V	V	V
PMA/ION (1ul) (Thermo Fisher Scientific)	X	X	X	V
Brefeldin A Solution (1ul) (Miltenyi Biotec, Germany)	V	V	V	V
GolgiStop (1ul) (BD Biosciences)	V	V	V	V

Table S6. rhAmpSeq panel design information

**Chromosome**	**amplicon start position**	**amplicon end position**	**guide start position**	**guide end position**
chr21	44729025	44729217	44729132	44729152
chr5	88484510	88484753	88484553	88484573
chr19	44260176	44260351	44260252	44260272
chr1	92412026	92412183	92412096	92412116
chr10	88827727	88827887	88827784	88827804
chr5	175472508	175472669	175472589	175472609
chr22	24761950	24762117	24762013	24762033
chr8	103180496	103180680	103180544	103180564
chr1	14947580	14947745	14947685	14947705
chr4	73914970	73915150	73915024	73915044
chr4	24450540	24450733	24450662	24450682
chr17	21322946	21323122	21322993	21323013
chr17	21764850	21765019	21764958	21764978
chr11	123221000	123221187	123221045	123221065
chrX	71111424	71111659	71111518	71111609
chr1	167730131	167730325	167730174	167730194

chr3	72764762	72764953	72764803	72764823
chr7	151485138	151485370	151485306	151485326
chr4	188581814	188582053	188581953	188581973
chr15	72016729	72016913	72016813	72016833
chr4	141210989	141211179	141211108	141211128
chr5	141657497	141657678	141657615	141657635
chr2	132703907	132704080	132704003	132704023

Table S7. Genomic coordinates and annotation of GUIDE-seq off-target sites

	**coordinates**	**Gene**	**#GUIDE-seq+A1:D24**
on-target	chrX:71111518-71111538	IL2RG	4875
OT1	chr1:167730174-167730194	MPZL1	3417
OT2	chr5:88484553-88484573	None	1329
OT3	chr7:151485306-151485326	RHEB	456
OT4	chr3:72764803-72764823	SHQ1	455
OT5	chr4:188581953-188581973	LINC01061	410
OT6	chr15:72016813-72016833	MYO9A	256
OT7	chr2:132704003-132704023	NCKAP5	213
OT8	chr5:141657615-141657635	ARAP3	200
OT9	chr4:141211108-141211128	RNF150	194
OT10	chr1:14947685-14947705	KAZN	139
OT11	chr4:73915024-73915044	None	136
OT12	chr4:24450662-24450682	PPARGC1A	116
OT13	chr19:44260252-44260272	ZNF233	104
OT14	chr17:21322993-21323013	None	83
OT15	chr1:92412096-92412116	None	72
OT16	chr17:21764958-21764978	None	58
OT17	chr5:175472589-175472609	None	54
OT18	chr10:88827784-88827804	ANKRD22	52
OT20	chr11:123221045-123221065	None	36
OT21	chr22:24762013-24762033	PIWIL3	21
OT22	chr8:103180544-103180564	BAALC	21
OT24	chr21:44729132-44729152	None	16

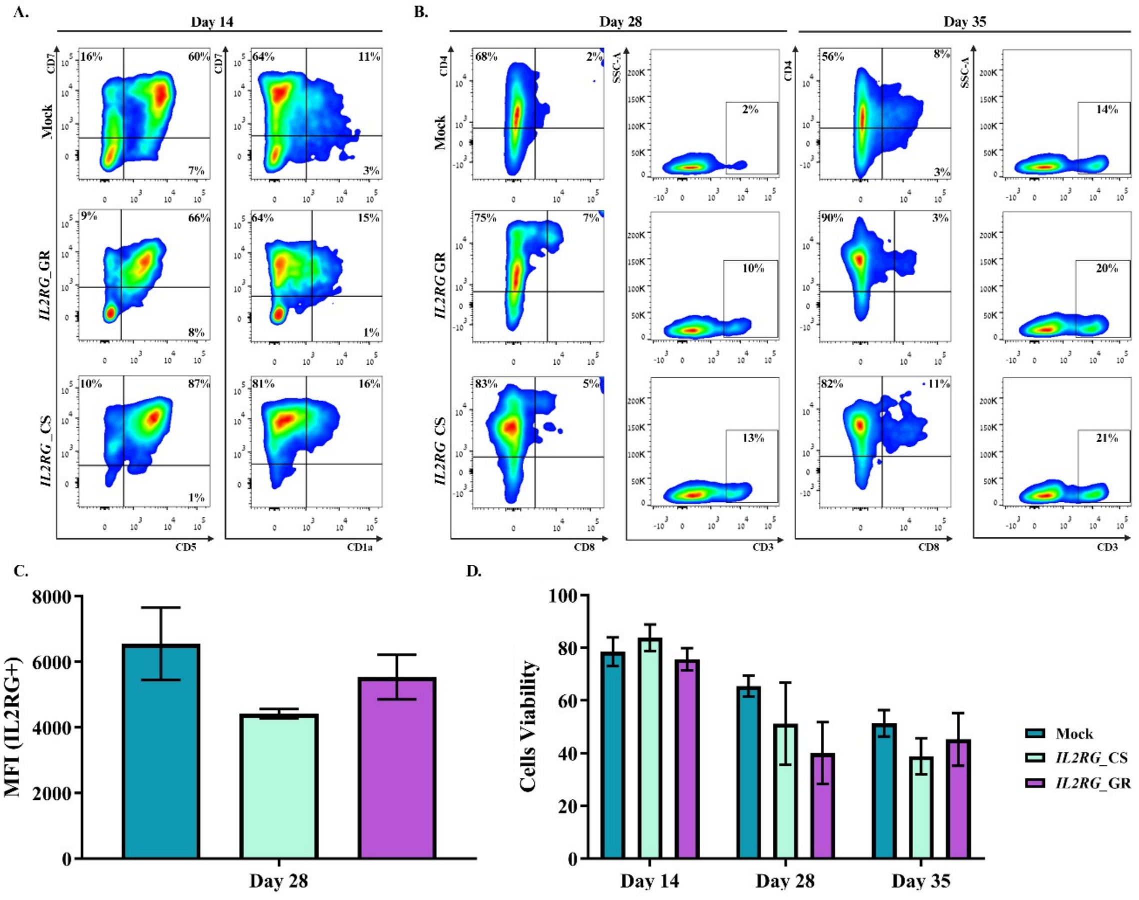

Figure S1: CS vs GR correction strategies: differentiation of edited CD34+ HSPCs into T lineages: (A) Representative FC plots from day 14 of IVTD. On day 14, the cells were tested for Pro T cell markers (CD7, CD5, and CD1a). Upper panel: Mock cells, Middle panel: *IL2RG*_GR, Lower panel: *IL2RG*_CS edited cells. **(B)** Representative FC plots from days 28 and 35 of the IVTD assay. On days 28 and 35, cells were tested for T-cell markers (CD3, CD4, and CD8). Upper panel, Mock cells; Middle panel, *IL2RG*_GR; Lower panel, *IL2RG*_CS edited cells. **(C)** MFI values of the *IL2RG* positive population analyzed by FC (N=3). **(D)** Viability analysis measured by FC on days 14, 28, and 35 of T cell differentiation. Mock (day 14, N = 5; day 28, N = 5; day 35, N =3), *IL2RG*_GR (day 14, N = 6; day 28, N = 6; day 35, N =4). Data are represented as mean ± SEM.

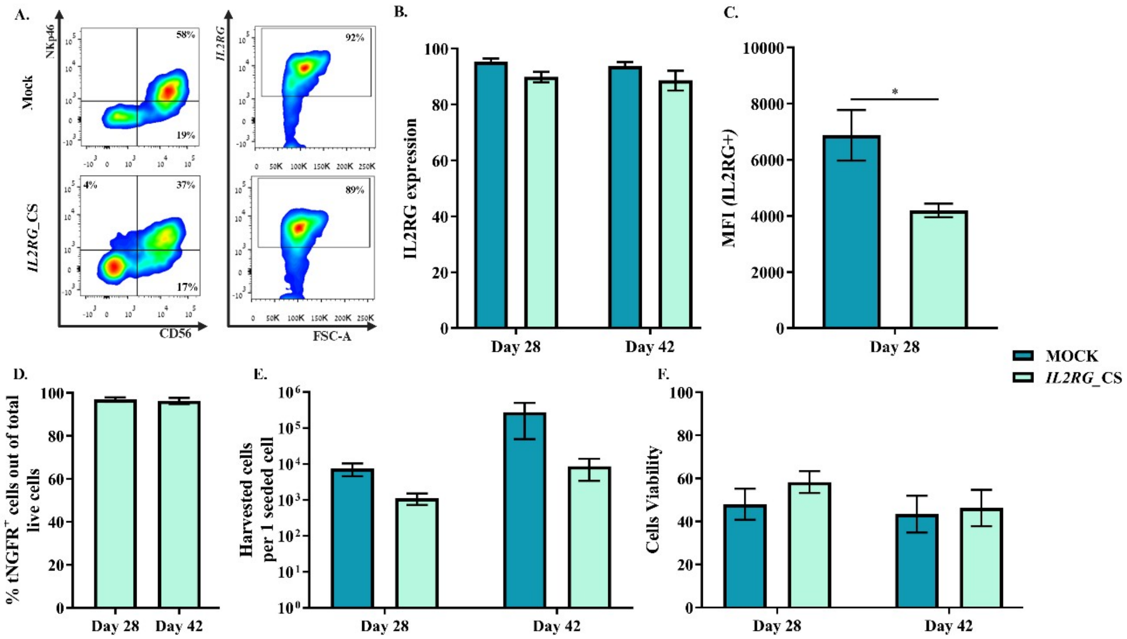

<u>Figure S2: Differentiation of edited CD34+ HSPCs into NK lineages</u> **(A)** Representative FC plots from day 42 of IVNKD assay showing CD56 and NKp46 NK markers. Upper panel: Mock cells, lower panel: *IL2RG*_CS edited cells. **(B)** IL2RG expression level during IVNKD. Mock (day 28, N = 7; day 42, N =5), *IL2RG*_CS (day 28, N = 7; day 42, N =5). **(C)** MFI values of IL2RG positive population analyzed by FC (N=3). t test * $p < 0.05$. **(D)** tNGFR expression levels during IVNKD were measured via FC. *IL2RG*_CS (day 28, N = 7; day 42, N =5) **(E)** Cell count on days 28 and 42 of IVNKD. Cell yield was calculated per 1 seeded cell into the IVNKD system, Mock (day 28, N = 7; day 42, N =4), *IL2RG*_CS (day 28, N = 6; day 35, N =4). **(F)** Viability analysis measured using FC on days 28 and 42 of IVNKD assay. Mock (day 28, N = 7; day 42, N =5), *IL2RG*_CS (day 28, N = 8; day 42, N =6). Data are represented as mean ± SEM.

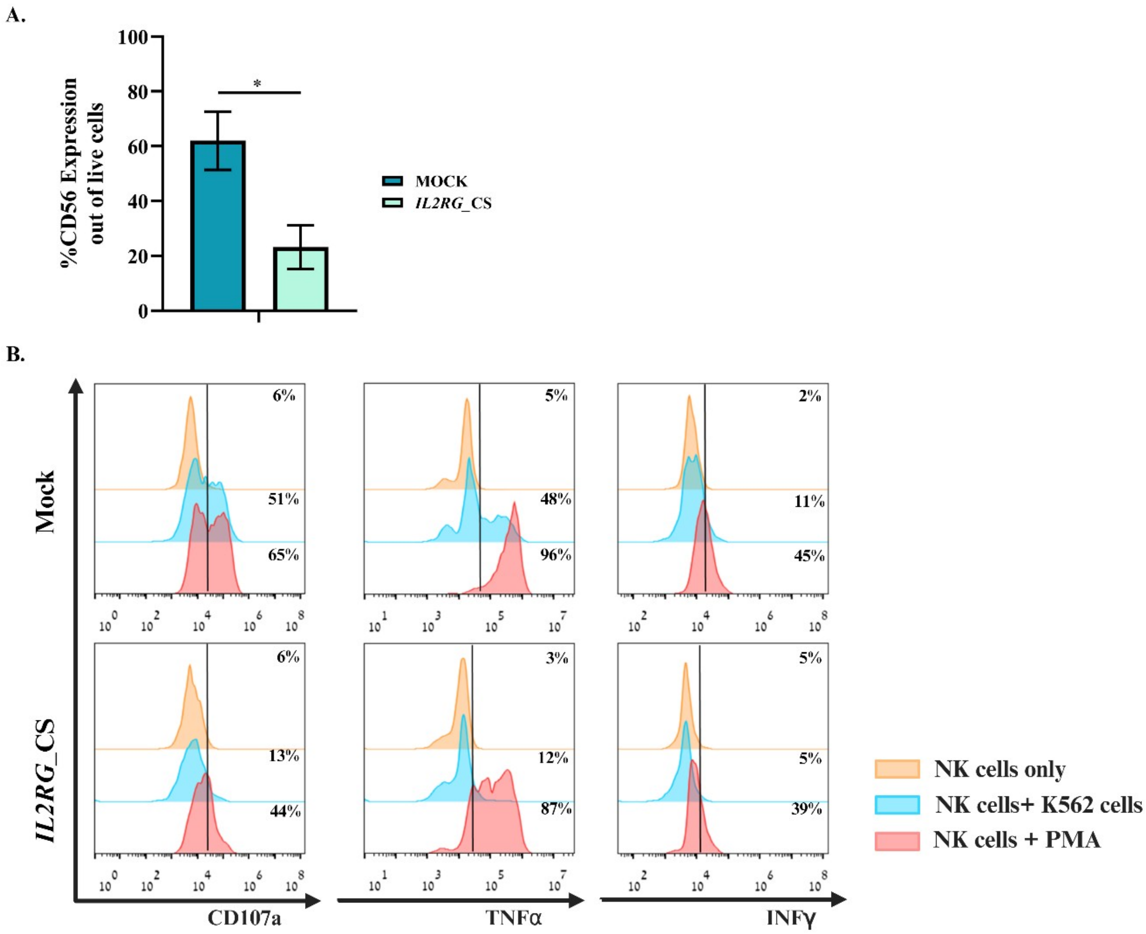

<u>Figure S3: Functionality of differentiated NK cells</u>: **(A)** CD56 expression levels of activated NK cells on day 42. Mock (N = 5), *IL2RG*_CS (N = 5), Data are represented as mean ± SEM. t test * $p < 0.05$. **(B)** Representative FC plots from functional assay on day 42 of NK cells co-cultured with K562 target cells/PMA, showing expression levels of CD107a, TNFα, and IFNγ (left, middle, and right plots, respectively). Upper panel, Mock cells; Lower panel, *IL2RG*_CS edited cells.

A.

Lymphocyte Subsets	Normal count	SCID-X1 patient 1	SCID-X1 patient 2
WBC/mm^3	5,000-13,000 cells/mm^3	8,900 cells/mm^3	4,610 cells/mm^3
Lymphocyte	3,400-7,600 cells/mm^3	1611 cells/mm^3	765 cells/mm^3
Lymphocyte % (out of WBC)	40%-80%	18.1%	16.6%
CD3	2,500-5,500 cells/mm^3	10 cells/mm^3	5 cells/mm^3
CD3 % (out of Lymphocyte)	60%-85%	0.6%	0.7%
CD4	436-1,394 cells/mm^3	10 cells/mm^3	5 cells/mm^3
CD4% (out of Lymphocyte)	36%-63%	0.7%	0.7%
CD8	166-882 cells/mm^3	11 cells/mm^3	8 cells/mm^3
CD8% (out of Lymphocyte)	15%-40%	0.7%	1%
CD20	50-300 cells/mm^3	1401 cells/mm^3	694 cells/mm^3
CD20% (out of Lymphocyte)	5%-25%	86%	91%
CD56+CD16+	6%-30%	8%	8%
TRECs	400	0	0

B.

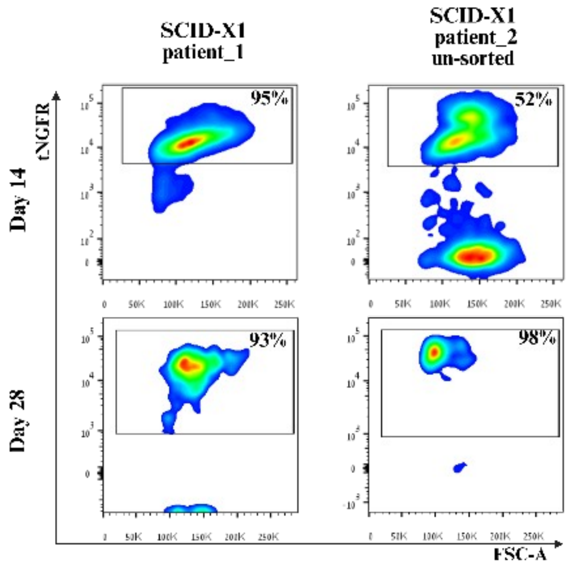

<u>Figure S4: SCID-X1 patient-derived HSPCs correction rescues T-cell differentiation</u> (A) Clinical data of c.865C>T SCID-X1 patient 1 and c.115+2_115+4del SCID-X1 patient_2 obtained from hematological test. **(B)** FC analysis of the editing cells based on tNGFR expressions on days 14 and 28 of IVTD. Left panel: SCID-X1 patient_1, right panel: patient SCID-X1 patient_2.

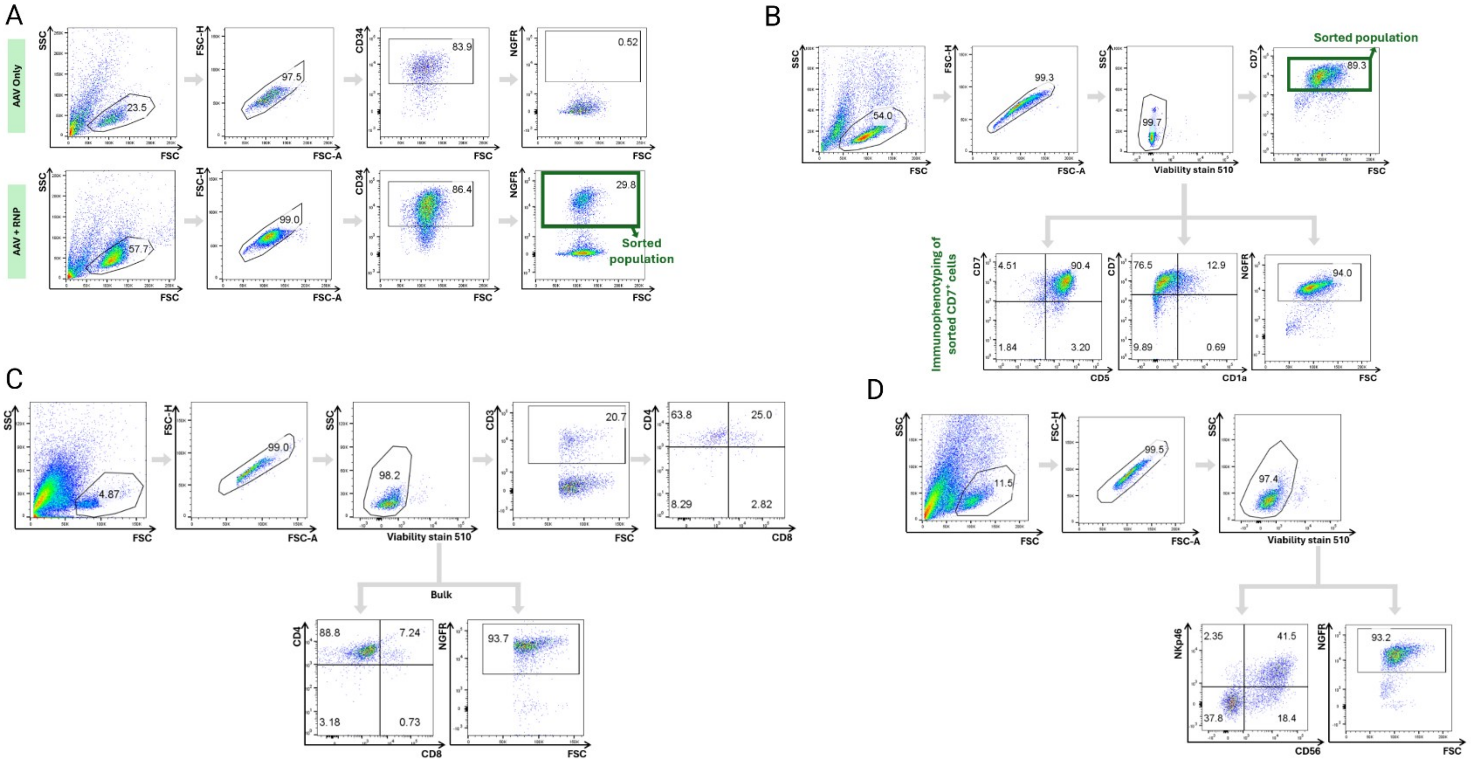

Figure S5. Gating and sorting strategies for analysis of hematopoietic differentiation following gene editing (CS2500, MOI).

(A) Sorting strategy at day 0 . The upper panels show the AAV-only control used to define gating thresholds. Cells were sequentially gated on FSC/SSC, singlets (FSC-H vs FSC-A), and CD34$^+$ cells, followed by NGFR expression. The same gating strategy was applied to AAV + RNP samples, and NGFR$^+$ cells were sorted (green gate). **(B)** Gating and sorting strategy at day 14 of differentiation. Cells were gated on FSC/SSC, singlets, and live cells (Viability stain 510$^-$), followed by selection of CD7$^+$ cells for sorting (green gate). Sorted CD7$^+$ cells were further characterized based on CD5 and CD1a expression, as well as NGFR expression. **(C)** Gating strategy for analysis of IVTD at day 28. Cells were sequentially gated on FSC/SSC, singlets, and live cells (Viability stain 510$^-$), followed by identification of CD3$^+$ cells. T cell subsets were further analyzed based on CD4 and CD8 expression. Bulk populations were additionally assessed for

CD4/CD8 distribution and NGFR expression. **(D)** Gating strategy for analysis of IVNKD at day 28. Cells were gated on FSC/SSC, singlets, and live cells (Viability stain 510^{-}), followed by analysis of NK populations based on CD56 and NKp46 expression. NGFR expression was evaluated within the gated population. All panels represent a representative example from CS2500 at the indicated multiplicity of infection (MOI).
